# 947. Evaluating the Outcomes of Infectious Diseases Consultation on methicillin susceptible *Staphylococcus aureus* Bacteremia

**DOI:** 10.1093/ofid/ofac492.790

**Published:** 2022-12-15

**Authors:** Alina Viteri, Natalie Tucker, Sara Barnstable

**Affiliations:** HSHS St. John's Hospital, Springfield, Illinois; HSHS St. John's Hospital, Springfield, Illinois; Franciscan Health Indianapolis, Indianapolis, Indiana

## Abstract

**Background:**

In 2014, an institutional project was completed assessing outcomes of beta-lactams versus vancomycin in patients with MSSA bacteremia. This research led to the development and implementation of a new standard of care for *Staphylococcus aureus* bacteremia using a bundle set that included ID consultation. The purpose of this research is to evaluate the impact mandatory ID consultation had on patient outcomes since this bundle set was implemented at our institution.

**Methods:**

This was a quasi-experimental, single center study of adults with MSSA bacteremia admitted from April 1, 2018 to August 31, 2021. In the instance of patients with multiple admissions with MSSA bacteremia, data from only the first admission was included in the final analysis. Patients were excluded if they had polymicrobial bacteremia or expired within 48 hours of admission. Patients were identified by utilizing reports generated from the pharmacy clinical decision support software of patients with at least one blood culture positive for MSSA. Patients in this list were further screened through manual chart review for inclusion and exclusion criteria. The primary endpoint of this study is inpatient mortality in the setting of MSSA bacteremia with and without an ID consultation. Inpatient mortality was defined as death by any cause during hospital admission. Secondary endpoints included time to bacterial clearance, ID consultation, and mortality.

**Results:**

Of the patients included, only 42 percent of patients in the pre-bundle set group were treated with MSSA targeted therapy (beta-lactam), in comparison to 86 percent in the post-bundle set group. The difference in secondary outcomes was not statistically significant. For the primary outcome of inpatient all-cause mortality, a 10 percent difference was found between the pre and post-bundle set groups, 18% vs. 8% (p = 0.01). The multivariate regression analysis also confirmed that the presence of an ID consult had a significant relationship with decreased mortality (p = 0.01).

**Conclusion:**

The all-cause mortality rate of adult patients with MSSA bacteremia significantly decreased after implementation of the *Staphylococcus aureus* bacteremia bundle set requiring an ID consultation.

**Disclosures:**

**All Authors**: No reported disclosures.

